# Consumption of critical nutrients and sweeteners related to the risk of chronic diseases in the population of Antioquia, according to the degree of food processing

**DOI:** 10.11606/s1518-8787.2024058005424

**Published:** 2024-07-22

**Authors:** Liliana Gaviria-Salinas, Juan Fernando Saldarriaga-Franco, Laura Inés González-Zapata, Gustavo Cediel

**Affiliations:** I Universidad de Antioquia Facultad Nacional de Salud Pública Escuela de Nutrición y Dietética Medellín ANT Colombia Universidad de Antioquia. Facultad Nacional de Salud Pública. Escuela de Nutrición y Dietética. Medellín, ANT, Colombia; II Universidad de Antioquia Facultad Nacional de Salud Pública Grupo de Investigación en Epidemiología Medellín ANT Colombia Universidad de Antioquia. Facultad Nacional de Salud Pública. Grupo de Investigación en Epidemiología. Medellín, ANT, Colombia; III Universidad de Antioquia Escuela de Nutrición y Dietética Grupo de Investigación en Determinantes Sociales y Económicos de la Salud y la Nutrición Medellín ANT Colombia Universidad de Antioquia. Escuela de Nutrición y Dietética. Grupo de Investigación en Determinantes Sociales y Económicos de la Salud y la Nutrición. Medellín, ANT, Colombia; IV Universidad de Antioquia Escuela de Nutrición y Dietética Grupo Saberes Alimentarios Medellín ANT Colombia Universidad de Antioquia. Escuela de Nutrición y Dietética. Grupo Saberes Alimentarios. Medellín, ANT, Colombia

**Keywords:** Processed Foods, Ultra-processed Products, Food Processing, Nova, Nutrient Profile

## Abstract

**OBJECTIVE:**

To analyze the consumption of critical nutrients and other sweeteners, according to the degree of food processing in the population of Antioquia.

**METHODS:**

Cross-Sectional Study. The dietary intake of 4,382 participants of the *Perfil Alimentario y Nutricional de Antioquia 2019* (Antioquia Food and Nutrition Profile 2019) was evaluated. Processed foods (PF) and ultra-processed products (UPP) reported by 24-hour recall were classified according to the Nova system. The Nutrient Profile Model (NPM) of the Pan American Health Organization (PAHO) was used. The amount of PF and UPP consumed with excess of critical nutrients related to chronic diseases (CD) was measured. The difference in average intake, the prevalence of excess intake, and the likelihood of inadequacy between groups with and without excess dietary content were assessed.

**RESULTS:**

Nearly 50% of the PF and UPP consumed had excess in at least one critical nutrient. The population consumed daily one or more products with excess in free sugar (73.3%), total fat (75.2%), saturated fat (77.0%), sodium (83.9%), and/or sweeteners (36.8%). Those who consumed products with excessive amounts had a higher intake of total fat (> 5.8%); saturated fat (> 3.8%); and sodium (> 698.7 mg) in adults and adolescents, in children 5–10 years (> 659.2 mg), and in children under 5 years (> 498 mg). Those who consumed products with excessive amounts presented the greatest possibilities of dietary inadequacy.

**CONCLUSION:**

The population of Antioquia that consumes PF and UPP with excessive amounts of free sugars, total fat, saturated fat, sodium, and/or sweeteners presents an unbalanced diet. Reducing the consumption of these products and returning to a natural and/or minimally processed diet may be an effective strategy to achieve the nutrient intake recommendations prioritized by PAHO in the population of Antioquia.

## INTRODUCTION

The traditional dietary pattern associated with healthy eating is being displaced by the increase in the supply of processed foods (FF) and ultra-processed products (UPP)^1^. In recent decades, obesity and other diet-related chronic diseases (CD) have increased in different age groups, becoming the leading cause of death and disability in the Americas region^2 , 3^.

Studies indicate that the consumption of UPPs favors the onset of obesity^4 , 5^, CD^6 , 7^, and the risk of all-cause mortality^8 - 11^. The Pan American Health Organization (PAHO) indicates that sales of UPPs have increased in Latin America, with an increase of 7.7% in Colombia^12^. Studies carried out in various countries have found that UPP consumption represents 5.9% of total energy intake in Colombia^13^, 20.4% in Brazil^14^, 28.6% in Chile^15^, and 58.5% in the United States^16^. In Antioquia, in the Food and Nutrition Profile (PANA 2019), it was found that 57.0% of the total average energy consumed by individuals comes from natural or minimally processed foods, 19.0% from culinary ingredients, and, between 9.0% to 15.0% comes from PF or UPPs^17^.

The PAHO Nutrient Profile Model (NPM) indicates the nutrients that should be analyzed, and indicates the maximum acceptable levels of consumption, establishing its application to industrially processed products that have been related to CD (PF, UPP). The nutrients it proposes to evaluate are: sodium, total fat, saturated fat, trans fat, and free sugars^18^. It also includes “other sweeteners” as food additives, which may be present in sweet-tasting foods, with or without added sugar, that are commonly consumed and promote the intake of more sweet foods and beverages, including those with sugar^18^.

The above suggests the need to study in the population of Antioquia: the profile of critical nutrients and the presence of other sweeteners in the PF and UPP consumed; the consumption of critical nutrients, according to the degree of food processing; and the contribution of PF and UPP to the excessive consumption of critical nutrients and other sweeteners using the PAHO Nutrient Profile Model.

## METHODS

In this cross-sectional study, the food consumption database, compiled as part of the Antioquia Food and Nutrition Profile (PANA 2019), conducted by the School of Nutrition and Dietetics of the University of Antioquia, was considered as a secondary source of information. The primary source of information was the nutritional labels of the PFs and UPPs described in the PANA 2019.

Food consumption data were collected on the basis of two 24-hour dietary reminders administered by interviewers (dietitian nutritionists) trained and standardized in the technique, who recorded the type of food, the name of the preparation, the ingredients and the amount of food consumed by the interviewee; whenever possible, the person responsible for food preparation was present. Food models were used to help participants better estimate the amount and weight of food consumed. The food models have been previously designed, validated, adjusted, and used in different studies. Data monitoring and critique was performed on 100% of the forms to verify that the data collection technique was carried out properly and that the quality of the data met the established standards^17^.

The first reminder was applied to 4,382 people, while the second reminder was applied to 1,215 people distributed in age groups, carried out on non-consecutive days and with an intermediate space of no more than seven days to adjust for intra-individual variability^17^. In the case of our study, only the information from the first recall was used, because the average consumption of the population was estimated for the critical nutrients (sodium, total fat, saturated fat, trans fat, free sugars, and sweeteners), and not the usual consumption, which requires at least two 24-hour recalls. The foods, products and preparations reported were classified in one of the four groups of the Nova food classification system. The aspects of the classification for Colombia are described in detail at^19^. Then, the sociodemographic information (age, age group, sex, educational level, and area of residence) and the consumption information of the individuals was extracted from the original database and reviewed in detail.

To obtain the information on critical nutrients, we proceeded as follows: 1) we took the data contained in the Colombian Food Composition Tables (TCA), such as the one from the *Instituto Colombiano de Bienestar Familiar* (ICBF). In the case of culinary ingredients, PF and UPP (as described in the PANA 2019), if there was a specific brand, we tried to obtain the information directly from the nutritional label or the web and, if necessary, we purchased the product; 2) in case of not finding the required data in the TCA and/or labels, homologation of the data not found was performed, taking the data of the required nutrient of the same food from other sources; and 3) when the previous option was not possible, imputation of the missing data with nutritionally similar food items was performed.

For the case of artificial non-caloric sweeteners (aspartame, sucralose, saccharin, and acesulfame potassium), natural non-caloric sweeteners (stevia), and/or caloric sweeteners (sorbitol, mannitol, lactitol, and isomalt), we proceeded as follows: the PFs and UPPs were taken (as they were described in the PANA 2019); if a specific brand was available, we tried to obtain the label or packaging on the web and in the necessary cases we made the purchase of the product. Then, the list of ingredients of the different PFs and UPPs was checked on the labels or packaging for the presence or absence of sweeteners.

Following the indications of the PAHO NPM, the amount of each nutrient per 100 grams or milliliters of edible or drinkable portion was estimated in the PF and UPP, in order to determine the excess of critical nutrients in each product, as follows:

- **Excessive amount of sodium:** if the ratio between the amount of sodium (mg) in any given amount of the product and the energy (kcal) is equal to or greater than 1:1 or greater than or equal to 1mg of sodium per 1kcal.
- **Excessive amount of free sugars:** if in any given quantity of the product, the amount of energy (kcal) from free sugars (grams of free sugars per 4kcal) is equal to or greater than 10.0% of the total energy (kcal).
- **Excessive amount of total fat:** if in any given quantity of the product, the amount of energy (kcal) from total fat (grams of total fat per 9kcal) is equal to or greater than 30.0% of the total energy (kcal).
- **Excessive amount of saturated fat:** if in any given quantity of the product, the amount of energy (kcal) from saturated fat (grams of saturated fat per 9kcal) is equal to or greater than 10.0% of the total energy (kcal).
- **Excessive amount of trans fat:** if in any given quantity of the product, the amount of energy (kcal) from trans fat (grams of trans fat per 9kcal) is equal to or greater than 1.0% of the total energy (kcal).
- **Sweeteners:** if the list of ingredients includes artificial or natural non-caloric sweeteners or caloric sweeteners (polyols)^18^.

### Statistical Analysis

The proportion of PF and UPP consumed by the population of Antioquia was calculated then, it was identified which proportion of products of each of these groups was excessive in the critical nutrients according to the PAHO NPM. Subsequently, the proportion of the population that presented consumption of 0, 1, 2, 3, and 4 or more PF and/or UPP, which contained at least one critical nutrient in excess, was determined.

At the same time, the average energy intake content of each critical nutrient was calculated, as well as the sodium intake in mg in the total population and in two fractions of the population: fraction of the population that consumed products with excessive content of critical nutrients and population that consumed products without excessive content of critical nutrients, according to PAHO NPM. Also, the prevalence of intake of critical nutrients was estimated, according to the WHO intake targets with their respective 95% confidence intervals, for the total population and the two fractions of the population.

For the univariate and bivariate descriptive analysis of the products consumed and the sociodemographic variables, absolute and relative distributions were used (categorical variables), and summary measures such as mean, median, standard deviation, quartiles, and minimum and maximum values (quantitative variables). The homoscedasticity assumption was tested for between-group variance. The Hosmer-Lemeshow criterion (p < 0.25) was applied to identify candidate variables to enter the explanatory models. Next, simple linear regression models were constructed with each of the independent variables. Then, multiple linear regression models were constructed, evaluating the significance of each independent variable (age group, area of residence, educational level, and sex) and the goodness of fit of the general model.

Subsequently, prevalence ratios were estimated by comparing the proportions of the population and groups that did not meet the WHO nutrient intake target, using logistic regression models adjusted for sociodemographic variables, in order to evaluate: the contribution to the consumption of products with excess of critical nutrients (according to the PAHO NPM); the inadequacy in the consumption of these nutrients; and the probability of intake of critical nutrients above the targets recommended by the WHO.

Analyses were performed in the statistical program Stata V.15. The project was approved by the Research Ethics Committee of the *Facultad Nacional de Salud Pública* of the *Universidad de Antioquia* (Act: 21030002-0066-2022 of 2022).

## RESULTS

The mean age of the population was 34.1 years (SD = 25.3). Of those surveyed, 56.5% were female, 62.3% were over 18 years, 34.4% had primary school education, and 27.8% had no education at all. 65.0% resided in urban areas ( Table 1 ).

**Table 1:** Demographic characteristics of the population of Antioquia 2019.

Indicator	Distribution	

	n	%
Age, mean (SD)	34.1 (25.3)	
Sex		
Male	1,905	43.5
Female	2,477	56.5
Age groups		
Early childhood: 0 to 5 years	409	9.3
Childhood: 6 to 11 years old	570	13.0
Adolescence: 12 to 17 years old	674	15.4
Youth: 18 to 26 years old	536	12.2
Adulthood: 27 to 59 years old	1,246	28.4
Old age: 60 years and older	947	21.6
Educational level		
Nursery, preschool	527	12.1
Completed elementary school (1st through 5th grade)	1,497	34.4
Secondary (grades 6 to 11)	744	17.1
Technical and/or technological (2 to 3 years)	275	6.3
University and/or postgraduate degree	104	2.4
None	1,211	27.8
Area of residence		
Urbana	2,847	65.0
Rural	1,535	35.0

SD: standard deviation

Of the total food and products consumed by the Antioquian population in 2019 (n = 1002) 47.3% were natural foods, minimally processed, or culinary ingredients, 13.9% of the products were processed, and 38.8% ultra-processed (data not shown). When the analysis was performed on 100 grams/milliliters of each product, it was identified that 50.0% of the PFs had a free sugar content of 5.0 gr or less, while for the UPPs it was 9.6 gr or less. In the case of total fat, saturated fat, and trans fat, 50.0% of the PF had a content of 5.7 gr, 2.6 gr, and 0.0 gr, respectively, while in the UPPs these values were 9.4 gr, 3.3 gr, and 0.0 gr, respectively ( Table 2 ).

**Table 2:** Amount of critical nutrients in 100 grams of product, according to Nova classification.

Nova Classiffication	Free sugar (gr)	Total fat (gr)	Saturated fat (gr)	Trans fat (gr)	Sodium (mg)	Sweetener (quantity)
Processed foods						
Media	11.9	10.9	5.1	0.1	436.3	0.0
SD	15.3	11.8	6.2	0.4	594.6	0.2
Min	0.0	0.0	0.0	0.0	0.0	0.0
Q1	0.2	2.0	0.0	0.0	37.5	0.0
Median	5.0	5.7	2.6	0.0	359.4	0.0
Q3	18.8	20.6	8.8	0.0	500.0	0.0
Max	66.7	52.5	23.3	3.3	4,333.3	1.0
IR	18.6	18.6	8.8	0.0	462.5	0.0
Ultra-processed products						
Media	19.6	12.6	5.8	0.0	581.4	0.3
SD	22.6	15.4	7.7	0.1	1,576.7	0.8
Min	0.0	0.0	0.0	0.0	0.0	0.0
Q1	1.6	0.0	0.0	0.0	50.0	0.0
Median	9.6	9.4	3.3	0.0	300.0	0.0
Q3	31.3	18.3	8.3	0.0	650.0	0.0
Max	100.0	100.0	50.0	1.2	19,272.7	3.0
IR	29.6	18.3	8.3	0.0	600.0	0.0

gr: grams; mg: milligrams; SD: standard deviation; IR: interquartile range.

When analyzing the sodium content, it was found that the PFs had a median of 359.4 mg in 100 grams of product, while the UPPs had a median of 300.0 mg. As for the amount of sweeteners in these products, it was found that the average in the PFs was 0.03 (min 0, maxx1) and in the UPPs 0.3 (min 0, max 3) ( Table 2 ).

Following the PAHO NPM criteria, it was found that of the total PF and UPPs, 62.8% presented an excessive amount of free sugar; 51.8% of total fat; 57.8% of saturated fat; 5.1% of trans fat; 45.3% of sodium; and 14.2% had sweeteners. Likewise, when analyzing the excess of each of these nutrients according to the Nova classification, it was found that 65.8% of the UPPs had an excess of free sugar, 53.4% of total fat, 59.0% of saturated fat, 5.1% of trans fat, 43.7% of sodium, and 18.6% contained sweeteners (data not shown).

The majority of the population consumed daily at least one PF and/or UPP, identified as excessive in some critical nutrient related to CD (according to the PAHO NPM), as follows: 73.3% with excessive content in free sugar, 75.2% in total fat, 77.0% in saturated fat, 8.4% in trans fat, 83.9% in sodium, and 36.8% contained sweeteners ( Table 3 ).

**Table 3:** Prevalence of consumption of products defined as excessive in nutrients prioritized by PAHO according to NPMa.

Type of products and food	Free Sugar		Total Fat		Saturated Fats		Trans Fats		Sodium		Sweeteners	
					
	n	% (95% CI)	n	% (95% CI)	n	% (95% CI)	n	% (95% CI)	n	% (95% CI)	n	% (95% CI)
Only foods that do not apply to the PAHO NPM^b^	330	7.5 (6.8–8.4)	330	7.5 (6.8–8.4)	330	7.5 (6.8–8.4)	330	7.5 (6.8–8.4)	330	7.5 (6.8–8.4)	330	7.5 (6.8–8.4)
Products defined as not excessive in critical nutrients related to NCDs according to the PAHO NPM^c^	839	19.2 (18.0–20.3)	759	17.3 (16.2–18.5)	679	15.5 (14.5–16.6)	3,68	84.0 (82.9–85.1)	376	8.6 (7.8–9.5)	2,442	55.7 (54.3–57.2)
Products defined as excessive in critical nutrients related to NCDs according to the PAHO NPM^d^												
1 product	1,020	23.3 (22.1–24.6)	1,102	25.2 (23.9–26.5)	1,008	23.0 (21.8–24.3)	311	7.1 (6.4–7.9)	1,113	25.4 (24.1–26.7)	461	10.5 (9.7–11.5)
2 products	796	18.2 (17.1–19.3)	841	19.2 (18.1–20.4)	864	19.7 (18.6–20.9)	51	1.2 (0.1–1.5)	965	22.0 (20.8–23.3)	467	10.7 (9.8–11.6)
3 products	559	12.8 (11.8–13.8)	606	13.8 (12.8–14.9)	621	14.2 (13.2–15.2)	7	0.2 (0.1–0.3)	661	15.1 (14.1–16.2)	328	7.5 (6.7–8.3)
≥ 4 products	838	19.1 (18.0–20.3)	744	17.0 (15.9–18.1)	880	20.1 (18.9–21.3)	1	0.0 (0.0–0.2)	937	21.4 (20.2–22.6)	354	8.1 (7.3–8.9)

95% CI: 95% confidence interval; CD: chronic diet-associated diseases; NPM-PAHO: Pan American Health Organization Nutrient Profile Model.

^a^ Food consumption. *Perfil Alimentario y Nutricional de Antioquia 2019* . n = 4,382 people.

^b^ Foods that do not apply to the PAHO NPM: Natural or minimally processed foods and processed culinary ingredients.

^c^ Foods that apply to the PAHO NPM, but without high content of CD-related nutrients according to the PAHO NPM (for free sugars < 10% of total energy, for total fat < 30% of total energy, for saturated fat < 10% of total energy, for trans fat < 1% of total energy, for sodium < 1 mg per kcal).

^d^ Products with excessive NCD-related nutrient content according to PAHO NPM (for free sugars ≥ 10% of total energy, for total fats ≥ 30% of total energy, for saturated fats ≥ 10% of total energy, for trans fats ≥ 1% of total energy, for sodium ≥ 1 mg per kcal).

Regarding the consumption of PF and/or UPP without excessive content of critical nutrients, it was found that 19.2%, 17.3%, and 15.5% of people consumed products without excessive content of free sugars, total fat, and saturated fat, respectively. In the case of trans fat and sodium, the proportion of people who consumed products without excess was 84.0% and 8.6%, respectively. On the other hand, an even smaller proportion of Antioquians (7.5%) consumed only unprocessed and minimally processed foods and culinary ingredients ( Table 3 ).

When analyzing the caloric intake from critical nutrients, it was found that in the diet of the total population of Antioquia, 16.7% of the total calories came from free sugars, 27.1% from total fats, 11.5% from saturated fats, 0.21% from trans fats. The average sodium content in the total diet (without taking into account table salt consumption) was 897.6 mg in adults and adolescents, 983.2 mg in children 5–10 years old, and 840.5 mg in children under 5 years old. As for the amount of sweeteners, the average daily consumption was 0.98, being in all cases higher in the group consuming products with excessive content in critical nutrients, compared to those consuming products without excessive content ( Table 4 ).

**Table 4:** Average content of nutrients prioritized by PAHO according to the NPM in the total daily diet of the population and in two fractions of the populationa

Nutrient content of the diet	The entire population	Fractions of the population with a diet composed of products		Coef. (95% CI) unadjusted^d^	Coef. (95% CI) adjusted^e^

		Excessive content of nutrients prioritized by PAHO^b^	No excessive content of nutrients prioritized by PAHO^c^		
			
Dietary nutrient content	Mean (95% CI)	Mean (95% CI)	Mean (95% CI)		
Free sugar (% of total energy intake)	16.7 (16.3–17.0)	16.8 (16.4–17.1)	16.4 (15.6–17.1)	0.4 (-0.3–1.3)	0.8 (0.1–1.5)
Total fat (% of total energy intake)	27.1 (26.9–27.4)	28.7 (28.4–29.0)	22.45 (21.9–23.0)	6.2 (5.6–6.8)	5.8 (5.3–6.4)
Saturated fat (% of total energy intake)	11.5 (11.3–11.6)	12.4 (12.2–12.5)	8.42 (8.2–8.7)	3.9 (3.65–4.21)	3.8 (3.5–4.1)
Trans fat (% of total energy intake)	0.2 (0.2–0.2)	0.4 (0.3–0.4)	0.2 (0.2–0.2)	0.2 (0.2–0.2)	0.2 (0.2–0.2)
Sodium (mg) Adults and adolescents	897.6 (866.3–928.9)	1,029.7 (996.7–1,062.6)	290.2 (219.4–360.9)	739.5 (661.5–817.5)	698.7 (620.8–776.6)
Sodium (mg) Children between 5 and 10 years old	983.2 (922.1–1,044.3)	1,044.2 (983.2–1,105.3)	361.1 (166.3–555.9)	683.1 (479.0–887.3)	659.2 (452.4–865.9)
Sodium (mg) Children < 5 years	840.5 (788.5–892.6)	872.2 (820.2–924.1)	353.6 (149.7–557.4)	518.6 (308.3–728.9)	498.0 (289.0–707.0)
Sweeteners (quantity/day)	0.98 (0.93–1.03)	2.68 (2.59–2.77)	0	2.68 (2.61–2.75)	2.67 (2.60–2.74)

95% CI: 95% confidence interval; CD: chronic diet-associated diseases; NPM-PAHO: Pan American Health Organization Nutrient Profile Model.

^a^ Food consumption. *Perfil Alimentario y Nutricional de Antioquia 2019* . n = 4,382 people.

^b^ Products with excessive content of CD-related nutrients according to PAHO NPM (for free sugars ≥ 10% of total energy, for total fats ≥ 30% of total energy, for saturated fats ≥ 10% of total energy, for trans fats ≥ 1% of total energy, for sodium ≥ 1 mg per kcal).

^c^ Foods that do not apply to the PAHO NPM (natural or minimally processed foods and processed culinary ingredients) and foods to which the PAHO NPM applies, but which do not have excessive CD-related nutrient content (for free sugars < 10% of total energy, for total fat < 30% of total energy, for saturated fat < 10% of total energy, for trans fat < 1% of total energy, for sodium < 1 mg per kcal).

^d^ Unadjusted linear regression models.

^e^ Linear regression models adjusted for age group, sex, educational level, and area of residence.

When analyzing the crude and adjusted models (by sociodemographic variables) for each critical nutrient, it was found that there were significant differences in all of them, given that the coefficient of the average percentage (free sugar, total fat, saturated fat, and trans fat), amount consumed in mg (sodium), and amount/day (sweeteners) was higher in the group that consumed products with excess in critical nutrients ( Table 4 ).

Table 5 shows the fractions of the population according to the composition of the diet, as follows: population with a diet with products with excessive content in critical nutrients, versus those who did not consume these products. In the case of the population that consumed products with an excessive content of free sugar, saturated fat, total fat, sodium, and trans fat, it was found that 75.5%, 71.3%, 42.6%, 10.9%, and 3.5%, respectively, exceeded the recommended upper limit for these nutrients, while in the population with a diet without products with excessive content in critical nutrients the percentage of the population that did not meet the recommended intake was lower, with 63.7% for free sugar, 29.8% for saturated fat, and 19.4% for total fat.

**Table 5:** Prevalence of non-recommended intake levels of nutrients prioritized by PAHOa in the total population and by fractions of the population with a diet with and without products with excessive content of these critical nutrients according to PAHOb nutrient profiling model.

Critical nutrient related to CD	The entire population	Fractions of the population with a diet composed of products		PR (95% CI)^e^	

		With excessive content of nutrients prioritized by PAHO^c^	Without excessive content of nutrients prioritized by PAHO^d^		
			
	Percentage of individuals that exceeded the intake targets recommended by the WHO (%)			Raw	Adjusted^f^

	Mean (95% CI)	Mean (95% CI)	Mean (95% CI)		
Free sugar	72.3 (71.0–73.7)	75.5 (74.0–77.0)	63.7 (60.8–66.4)	1.2 (1.1–1.2)	1.15 (1.1–1.2)
Total fat	36.9 (35.4–38.3)	42.6 (41.0–44.3)	19.4 (17.1–21.8)	2.2 (1.9–2.5)	2.0 (1.7–2.2)
Saturated fats	61.7 (60.3–63.7)	71.3 (69.7–72.8)	29.8 (27.1–32.7)	2.4 (2.2–2.6)	2.2 (2.0–2.5)
Trans fats	0.7 (0.5–1.0)	3.6 (2.1–6.0)	0.4 (0.2–0.7)	8.8 (4.3–18.2)	8.2 (3.9–17.2)
Sodium	9.2 (8.4–10.1)	10.9 (9.9–11.9)	0.6 (0.2–1.5)	19.2 (7.2–51.3)	15.3 (5.7–40.9)
Sweeteners	36.7 (35.3–38.2)	.	.	1.6 (1.6–1.7)	1.6 (1.6–1.7)

95% CI: 95% confidence interval; CD: chronic diet-associated diseases; NPM-PAHO: Pan American Health Organization Nutrient Profile Model.

^a^ Critical nutrient content related to NCDs: free sugars, total fat, saturated fat, and trans fat (% of total energy intake). Sodium content: total sodium (mg) minus the recommended value per age group [2000 mg (for adults and adolescents); 1640mg (for children 5-10 years); and 1122 mg (for children under 5 years)]. Guideline: sodium intake for adults and children. World Health Organization; 2012; Human energy requirements. FAO/WHO/UN; 2004

^b^ Food consumption. *Perfil Alimentario y Nutricional de Antioquia 2019* . n = 4,382 people.

^c^ Products with excessive content of CD-related nutrients according to PAHO NPM (for free sugars ≥ 10% of total energy, for total fats ≥ 30% of total energy, for saturated fats ≥ 10% of total energy, for trans fats ≥ 1% of total energy, for sodium ≥ 1 mg per kcal).

^d^ Foods that do not apply to the PAHO NPM (natural or minimally processed foods and processed culinary ingredients) and foods to which the PAHO NPM applies, but which do not have excessive CD-related nutrient content (for free sugars < 10% of total energy, for total fat < 30% of total energy, for saturated fat < 10% of total energy, for trans fat < 1% of total energy, for sodium < 1 mg per kcal).

^e^ Coefficients of logistic regression models.

^f^ Adjusted for age groups, sex, educational level, and area of residence.

Likewise, Table 5 shows the adjusted prevalence ratios, indicating that the fraction of the population that consumed products with an excessive content of critical nutrients has a significant increase in the possibility of inadequacy in the consumption of free sugars, total fat, saturated fat, trans fat, sodium, and sweeteners when compared to the fraction of the population that did not consume products with an excessive content of critical nutrients (the prevalence ratio varies between 1.15 (95% CI 1.1–1.2) for free sugar and 15.3 (95% CI 5.7–40.9) for sodium.

## DISCUSSION

This research shows that nearly half of the PF and UPP consumed by the population of Antioquia had an excess of at least one critical nutrient. The results are similar to studies conducted in Colombia, Mexico, and Honduras^20^. Most of the subjects consumed one or more of these products daily. In addition, it should be noted that those who consumed PF and/or UPPs with excessive amounts of some critical nutrient had a diet with poorer nutritional quality and the greatest possibility of inadequacy in the consumption of critical nutrients and sweeteners related to CD (non-compliance with WHO intake recommendations).

On the other hand, a smaller proportion of individuals consumed products with excess trans fat, a situation that can be partly explained by the Colombian regulations for this component, such as: article 8 of law 1355 of 2009^23^, resolution 2508 of 2012^24^, and resolution 2154 of 2012, issued by the Ministry of Health and Social Protection^25^.

In line with the evidence, with the implementation of front-of-package warning labeling (based on the PAHO nutrient profile), most PFs and UPPs will be candidates to carry at least one label and/or be taxed with a health tax, in order to reduce their consumption and health consequences^26^.

The diets of people who consumed products with an excessive content of critical nutrients contained significantly more total fat (> 5.8%), more saturated fat (> 3.8%), and a higher amount of sodium (> 498 mg), compared to the diets of people who did not consume these products. The results agree with the research of Machado and collaborators^26^. In the case of free sugar, there were smaller differences, possibly because Antioquians have a high consumption of natural and/or minimally processed foods with high free sugar content such as: panela, honey, and natural juices with added sugar, which could indicate that the contribution of free sugar in the diet comes from different sources.

On the other hand, it is important to mention that the PAHO CPF also recommends warning when PF and UPPs contain any amount of sweeteners (artificial, natural, caloric, and non-caloric). Although the higher proportion of sweeteners consumed by individuals with PF and UPP intake was identified (2.7 sweeteners more), the exact amount of sweeteners consumed by the Antioquian population in their diet (grams or milligrams) is unknown, because the industry is not required to declare the amount (they only report their presence in the list of ingredients); likewise, the available evidence is not conclusive about their beneficial or harmful effects on health^29^.

This study showed that the intake of products with excessive content of critical nutrients explains the inadequacy in the diet of the population, according to WHO recommendations. This result is similar to those found in studies conducted in Australia^26^ and Uruguay^27 , 28^.

The present research supports the public policy measures established in the department of Antioquia, through the *Plan Docenal de Seguridad Alimentaria y Nutricional* (Docennial Plan for Food and Nutritional Security), in terms of reducing UPP consumption to less than 10.0% of total energy consumed by 2031^30^, as well as national policies and proposals to implement black octagonal front warning labeling (with the word excess), according to PAHO nutrient profiling recommendations^18^; it also gives solid bases to the implementation of healthy taxes on UPPs.

The strengths of this study include: The quality of the database and the significant number of records collected through the 24-hour recall, which was performed by dietitian nutritionists standardized in the technique; the representativeness of the sample (departmental level), by age groups, sex, educational level, and area of residence; the modeling of the differential possibilities of dietary inadequacy. This is the first study in Antioquia that evaluates the consumption of nutrients prioritized by PAHO, as well as the content of sweeteners in the diet, these results being the baseline for local and national public policy measures (regulation of PF and UPP).

Regarding limitations, our study was based on only a 24-hour recall, so it is possible that in some cases it does not represent the usual intake^17^. As for sodium intake, it is likely to be underestimated because the PANA did not include discretionary salt, added by individuals in foods prepared at home or in restaurants. On the other hand, the 24-hour reminder instrument used in the PANA does not inquire about the brand of PF and UPP consumed by each individual, which makes it difficult to know with full certainty the content of some of the critical nutrients (mainly free sugars, trans fats, and sweeteners, the latter in the list of ingredients) that are not included in the food composition tables; even so, this limitation was addressed, in the case of free sugars by applying panel D of the PAHO nutrient profiling model, and in the case of trans fats and sweeteners by tracing the information directly on the labels of products similar to those reported in the database and/or by consulting the USDA database.

## CONCLUSION

The population of Antioquia that consumes PF and UPPs with excessive amounts of free sugars, total fat, saturated fat, sodium, and/or sweeteners has an unbalanced diet. Reducing the consumption of these products and returning to a natural and/or minimally processed diet may be an effective strategy to achieve the nutrient intake recommendations prioritized by PAHO in the population of Antioquia.
