# Supplementary material for: Consumption of critical nutrients and sweeteners related to the risk of chronic diseases in the population of Antioquia, according to the degree of food processing
Source: Rev Saude Publica. 2024 Jul 22;58:31. doi: 10.11606/s1518-8787.2024058005424 (PMC11319031; doi:10.11606/s1518-8787.2024058005424)
Supplement: Translation

# Translation: Consumo de nutrientes críticos y edulcorantes relacionados con el riesgo de enfermedades crónicas en la población antioqueña, según grado de procesamiento de los alimentos

**Authors:** Liliana Gaviria-Salinas, Juan Fernando Saldarriaga-Franco, Laura Inés González-Zapata, Gustavo Cediel

**Affiliations:** I Colombia Universidad de Antioquia. Facultad Nacional de Salud Pública. Escuela de Nutrición y Dietética. Medellín, ANT, Colombia; II Colombia Universidad de Antioquia. Facultad Nacional de Salud Pública. Grupo de Investigación en Epidemiología. Medellín, ANT, Colombia; III Colombia Universidad de Antioquia. Escuela de Nutrición y Dietética. Grupo de Investigación en Determinantes Sociales y Económicos de la Salud y la Nutrición. Medellín, ANT, Colombia; IV Colombia Universidad de Antioquia. Escuela de Nutrición y Dietética. Grupo Saberes Alimentarios. Medellín, ANT, Colombia

**Keywords:** Alimentos Procesados, Productos Ultraprocesados, Procesamiento de Alimentos, Nova, Perfil de Nutrientes

## Abstract

**OBJETIVO:**

Analizar el consumo de nutrientes críticos y otros edulcorantes, según el grado de procesamiento de los alimentos en la población Antioqueña.

**MÉTODOS:**

En este estudio transversal, se evaluó el consumo alimentario de 4,382 participantes del Perfil Alimentario y Nutricional de Antioquia 2019. Los alimentos procesados (AP) y productos ultraprocesados (PUP) informados por recordatorio de 24 horas se clasificaron según sistema Nova. Se usó el Modelo de Perfil de Nutrientes (MPN) de la Organización Panamericana de la Salud (OPS). Se midió la cantidad de AP y PUP consumidos con exceso en nutrientes críticos, relacionados con las enfermedades crónicas (EC). Se evaluó la diferencia en el consumo promedio, la prevalencia del consumo excesivo, y las posibilidades de inadecuación entre los grupos que tenían una dieta con y sin contenido excesivo.

**RESULTADOS:**

Cerca del 50% de los AP y PUP consumidos presentaba exceso en al menos un nutriente crítico. La población consumió diariamente uno o más productos con exceso en azúcar libre (73.3%), grasa total (75.2%), grasa saturada (77.0%), sodio (83.9%) y/o edulcorantes (36.8%). Quienes consumían productos con cantidades excesivas tenían una mayor ingesta de grasas totales (> 5.8%); grasas saturadas (> 3.8%); y sodio (> 698.7 mg) en adultos y adolescentes, en niños de 5–10 años (> 659.2 mg), y en menores de 5 años (> 498 mg). Quienes consumieron productos con cantidades excesivas presentaron las mayores posibilidades de inadecuación en la dieta.

**CONCLUSIÓN:**

La población antioqueña que consume AP y PUP con cantidades excesivas de azúcares libres, grasas totales, grasas saturadas, sodio y/o edulcorantes, presenta una dieta desbalanceada. Reducir el consumo de estos productos y volver a una dieta natural y/o mínimamente procesada puede ser una estrategia efectiva para alcanzar las recomendaciones de ingesta de nutrientes priorizados por OPS en la población antioqueña.

## INTRODUCCIÓN

El patrón alimentario tradicional asociado con una alimentación saludable se viene desplazando por el aumento en la oferta de alimentos procesados (AP) y productos ultraprocesados (PUP)^1^. En las últimas décadas, la obesidad y otras enfermedades crónicas (EC) asociadas a la alimentación se han incrementado en diferentes grupos etarios, convirtiéndose en la principal causa de muerte y discapacidad en la región de las Américas^2 , 3^.

Estudios indican que el consumo de PUP favorece la aparición de la obesidad^4 , 5^, EC^6 , 7^, y el riesgo de mortalidad por todas las causas^8 - 11^. La Organización Panamericana de la Salud (OPS), señala que en América Latina se han incrementado las ventas de PUP, refiriendo un aumento del 7.7% en Colombia^12^. Estudios realizados en diversos países, han encontrado que el consumo de PUP representa el 5.9% de la ingesta total de energía en Colombia^13^, el 20.4% en Brasil^14^, el 28.6% en Chile^15^, y el 58.5% en Estados Unidos^16^. En Antioquia, en el Perfil Alimentario y Nutricional (PANA 2019), se encontró que el 57.0% de la energía promedio total consumida por los individuos proviene de los alimentos naturales o mínimamente procesados, el 19.0% de los ingredientes culinarios, y entre el 9.0% y 15.0% proviene de los AP o PUP^17^.

El Modelo de Perfil de Nutrientes (MPN) de la OPS señala los nutrientes que deben ser analizados, e indica los niveles máximos aceptables de consumo, estableciendo su aplicación a los productos procesados industrialmente que se han relacionado con las EC (AP, PUP). Los nutrientes que propone evaluar son: sodio, grasas totales, grasas saturadas, grasas trans y azúcares libres^18^. Asimismo, incluye “otros edulcorantes” como aditivos alimentarios, que pueden estar presentes en alimentos de sabor dulce, con adición o no de azúcar, que son de consumo habitual y promueven la ingesta de más alimentos y bebidas dulces, incluidos los que tienen azúcar^18^.

Lo anterior sugiere la necesidad de estudiar en la población Antioqueña: el perfil de nutrientes críticos y la presencia de otros edulcorantes en los AP y PUP consumidos; el consumo de nutrientes críticos, según el grado de procesamiento de los alimentos; y la contribución de los AP y PUP al consumo excesivo de nutrientes críticos y otros edulcorantes empleando el Modelo de Perfil de Nutrientes de la OPS.

## MÉTODOS

En ese estudio transversal, se consideró como fuente de información secundaria la base de datos de consumo de alimentos, recopilada como parte del Perfil Alimentario y Nutricional de Antioquia (PANA 2019), realizado por la Escuela de Nutrición y Dietética de la Universidad de Antioquia. La fuente de información primaria fueron las etiquetas nutricionales de los AP y PUP descritos en el PANA 2019.

Los datos del consumo de alimentos se recopilaron sobre la base de dos recordatorios dietéticos de 24 horas administrados por entrevistadores (nutricionistas dietistas) capacitados y estandarizados en la técnica. Ellos registraron el tipo de alimento, el nombre de la preparación, los ingredientes y la cantidad de alimento consumida por el entrevistado; siempre que fuera posible estuvo presente la persona responsable de preparación de los alimentos. Fueron usados modelos de alimentos para ayudar a los participantes a una mejor estimación en la cantidad y el peso del alimento consumido. Los modelos alimentarios han sido previamente diseñados, validados, ajustados y empleados en diferentes estudios. Se realizó supervisión y crítica de los datos en el 100% de los formularios para verificar que la técnica de recolección de la información se llevaba a cabo adecuadamente y que la calidad de los datos cumpliera con los estándares establecidos^17^.

El primer recordatorio se aplicó a 4.382 personas, mientras que el segundo recordatorio se aplicó a 1.215 distribuidos en los grupos de edad, realizados en días no consecutivos y con un espacio intermedio no mayor a siete días para ajustar la variabilidad intra-individual^17^. Para el caso de nuestro estudio solo se empleó la información de primer recordatorio, debido a que se estimó el consumo promedio de la población en los nutrientes críticos (sodio, grasas totales, grasas saturadas, grasas trans, azúcares libres y edulcorantes), y no el consumo usual que si requiere de al menos dos recordatorios de 24 horas. Los alimentos, productos y preparaciones informados se clasificaron en uno de los cuatro grupos del sistema de clasificación de alimentos Nova. Los aspectos de la clasificación para Colombia, se encuentra descritos detalladamente^19^. Luego, de la base original se extrajo la información sociodemográfica (edad, grupo edad, sexo, nivel educativo y zona de residencia), y la información de consumo de los individuos, la cual fue revisada minuciosamente.

Para obtener la información de nutrientes críticos se procedió así: 1) se tomaron los datos consignados en las Tablas de Composición de Alimentos (TCA) colombianas como la del Instituto Colombiano de Bienestar Familiar (ICBF). En el caso de los ingredientes culinarios, AP y PUP (tal cual estaban descritos en el PANA 2019), si se tenía una marca específica, se procuró obtener la información directa de la etiqueta nutricional o la web (en los casos necesarios se realizó la compra del producto); 2) en caso de no encontrar los datos requeridos en las TCA y/o etiquetas, se realizó homologación de los datos no encontrados, tomando el dato del nutriente requerido del mismo alimento de otras fuentes; y 3) cuando no fue posible la opción anterior, se realizó imputación del dato faltante con ítems alimentarios nutricionalmente similares.

Para el caso de los edulcorantes artificiales no calóricos (aspartame, sucralosa, sacarina y potasio de acesulfamo), edulcorantes naturales no calóricos (estevia); y/o edulcorantes calóricos (sorbitol, manitol, lactitol e isomalt), se procedió de la siguiente manera: se tomaron los AP y PUP (tal cual estaban descritos en el PANA 2019); si se tenía una marca especifica, se procuró obtener la etiqueta o empaque en la web y en los casos necesarios se realizó la compra del producto. Luego se revisó en las etiquetas o empaques la lista de ingredientes de los diferentes AP y PUP la presencia o ausencia de edulcorantes.

Siguiendo las indicaciones del MPN de la OPS, en los AP y PUP, se estimó la cantidad de cada uno de los nutrientes por 100 gramos o mililitros de porción comestible o bebible, con el fin de poder determinar el exceso de nutrientes críticos en cada producto, así:

- **Cantidad excesiva de sodio:** si la razón entre la cantidad de sodio (mg) en cualquier cantidad dada del producto y la energía (kcal) es igual o mayor a 1:1 o sea, mayor o igual a 1 mg de sodio por 1 kcal.
- **Cantidad excesiva de azúcares libres:** si en cualquier cantidad dada del producto,
- la cantidad de energía (kcal) proveniente de los azúcares libres (gramos de azúcares libres por 4 kcal) es igual o mayor al 10.0% del total de energía (kcal).
- **Cantidad excesiva de grasas totales:** si en cualquier cantidad dada del producto,
- la cantidad de energía (kcal) proveniente del total de grasas (gramos de grasas totales por 9 kcal) es igual o mayor al 30.0% del total de energía (kcal).
- **Cantidad excesiva de grasas saturadas:** si en cualquier cantidad dada del producto, la cantidad de energía (kcal) proveniente de grasas saturadas (gramos de grasas saturadas por 9 kcal) es igual o mayor al 10.0% del total de energía (kcal).
- **Cantidad excesiva de grasas trans:** si en cualquier cantidad dada del producto,
- la cantidad de energía (kcal) proveniente de grasas trans (gramos de grasas trans por
- 9 kcal) es igual o mayor al 1.0% del total de energía (kcal).
- **Edulcorantes:** si en la lista de ingredientes incluye edulcorantes artificiales o naturales no calóricos o edulcorantes calóricos (polialcoholes)^18^.

### Análisis Estadístico

Se calculó la proporción de AP y PUP consumidos por la población antioqueña, luego, se identificó que la proporción de productos de cada uno de estos grupos fue excesiva en los nutrientes críticos según el MPN de la OPS. Posteriormente, se determinó la proporción de la población que presentó un consumo de 0, 1, 2, 3 y 4 o más AP y/o PUP, que contenía al menos un nutriente crítico en exceso.

Paralelamente, se calculó el contenido promedio de ingesta de energía de cada nutriente crítico, y la ingesta de sodio en mg en el total de la población y en dos fracciones de la población: la fracción de la población que consumía productos con contenido excesivo de nutrientes críticos y la población que consumía productos sin contenido excesivo de nutrientes críticos, según MPN de la OPS). También, se estimó la prevalencia de la ingesta de nutrientes críticos, de acuerdo con las metas de ingesta de la OMS con sus respectivos intervalos de confianza del 95%, esto para la población total y las dos fracciones de la población.

Para el análisis descriptivo univariado y bivariado de los productos consumidos y las variables sociodemográficas, se emplearon distribuciones absolutas y relativas (variables categóricas), y medidas de resumen como la media, mediana, desviación estándar, cuartiles y valores mínimos y máximos (variables cuantitativas). Se probó el supuesto de homocedasticidad para la varianza entre grupos. Se aplicó el criterio de Hosmer-Lemeshow (p < 0.25) para identificar las variables candidatas a ingresar en los modelos explicativos. Seguidamente, se construyeron modelos de regresión lineal simples con cada una de las variables independientes. Luego, se construyeron modelos de regresión lineal múltiple, evaluando significancia de cada variable independiente (grupo de edad, zona de residencia, nivel educativo y sexo) y la bondad de ajuste del modelo general.

Posteriormente, se estimaron las razones de prevalencia al comparar las proporciones de la población y los grupos que no cumplieron con el objetivo de ingesta de nutrientes de la OMS. Para esto, se emplearon modelos de regresión logística ajustados por las variables sociodemográficas, con el fin de evaluar: la contribución al consumo de productos con exceso de nutrientes críticos (según el MNP de OPS); la inadecuación en el consumo de estos nutrientes; y la probabilidad de la ingesta de los nutrientes críticos, por encima de las metas recomendadas por la OMS.

Los análisis se realizaron en el programa estadístico Stata V.15. El proyecto fue aprobado por el Comité de Ética de la Investigación de la Facultad Nacional de Salud Pública de la Universidad de Antioquia (Acta: 21030002-0066-2022 de 2022).

## RESULTADOS

La edad promedio de la población fue de 34.1 años (DE = 25.3). El 56.5% de los encuestados eran mujeres, 62.3% tenían más de 18 años, 34.4% tenían como nivel educativo la primaria, y 27.8% ningún tipo de educación. El 65.0% residían en zona urbana ( Tabla 1 ).

**Tabla 1:** Características demográficas de la población antioqueña 2019.

Indicador	Distribución	

	n	%
Edad, promedio (DE)	34.1 (25.3)	
Sexo		
Masculino	1,905	43.5
Femenino	2,477	56.5
Grupos de edad		
Primera infancia: 0 a 5 años	409	9.3
Infancia: 6 a 11 años	570	13.0
Adolescencia: 12 a 17 años	674	15.4
Juventud: 18 a 26 años	536	12.2
Adultez: 27 a 59 años	1,246	28.4
Vejez: 60 años en adelanta	947	21.6
Nivel educativo		
Sala cuna, guardería, preescolar	527	12.1
Primaria completa (1° a 5°)	1,497	34.4
Secundaria (6° a 11°)	744	17.1
Técnico y/o tecnológico (2 a 3 años)	275	6.3
Universidad y/o posgrado	104	2.4
Ninguno	1,211	27.8
Zona de residencia		
Urbana	2,847	65.0
Rural	1,535	35.0

DE: desviación estándar

Del total de alimentos y productos consumidos por la población antioqueña en 2019 (n = 1,002), 47.3% eran alimentos naturales, mínimamente procesados o ingredientes culinarios, 13.9% de los productos eran procesados y 38.8% ultraprocesados (datos no mostrados). Al realizar el análisis en 100 gramos/mililitros de cada producto, se identificó que el 50.0% de los AP tenían un contenido de azúcares libres de 5.0 gr o menos, mientras que para los PUP fue de 9.6 gr o menos. En el caso de las grasas totales, grasas saturadas y grasas trans, el 50.0% de los AP tenían un contenido de 5.7 gr, 2.6 gr y 0.0 gr, respectivamente, mientras que en los PUP estos valores fueron de 9.4 gr, 3.3 gr y 0.0 gr, respectivamente ( Tabla 2 ).

**Tabla 2:** Cantidad de nutrientes críticos en 100 gramos de producto, según clasificación Nova.

Clasificación Nova	Azúcar libre (gr)	Grasa total (gr)	Grasa saturada (gr)	Grasa trans (gr)	Sodio (mg)	Edulcorante (cantidad)
Alimentos procesados						
Media	11.9	10.9	5.1	0.1	436.3	0.0
DE	15.3	11.8	6.2	0.4	594.6	0.2
Min	0.0	0.0	0.0	0.0	0.0	0.0
Q1	0.2	2.0	0.0	0.0	37.5	0.0
Mediana	5.0	5.7	2.6	0.0	359.4	0.0
Q3	18.8	20.6	8.8	0.0	500.0	0.0
Máx	66.7	52.5	23.3	3.3	4,333.3	1.0
RI	18.6	18.6	8.8	0.0	462.5	0.0
Productos ultraprocesados						
Media	19.6	12.6	5.8	0.0	581.4	0.3
DE	22.6	15.4	7.7	0.1	1,576.7	0.8
Min	0.0	0.0	0.0	0.0	0.0	0.0
Q1	1.6	0.0	0.0	0.0	50.0	0.0
Mediana	9.6	9.4	3.3	0.0	300.0	0.0
Q3	31.3	18.3	8.3	0.0	650.0	0.0
Máx	100.0	100.0	50.0	1.2	19,272.7	3.0
RI	29.6	18.3	8.3	0.0	600.0	0.0

gr: gramos; mg: miligramos; DE: desviación estándar; RI: rango intercuartílico.

Al analizar el contenido de sodio, se encontró que los AP tenían una mediana de 359.4 mg en 100 gramos de producto, mientras que los PUP tenían una mediana de 300.0 mg. En cuanto a la cantidad de edulcorantes en estos productos, se halló que el promedio en los AP fue de 0.03 (min 0, máx1) y en los PUP de 0.3 (min 0, máx 3) ( Tabla 2 ).

Siguiendo los criterios del MPN de la OPS, se encontró que del total de AP y PUP, el 62.8% presentaron una cantidad excesiva de azúcar libre; 51.8% de grasa total; 57.8% de grasa saturada; 5.1% de grasa trans; 45.3% de sodio, y 14.2% tenían edulcorantes. Así mismo, al analizar el exceso de cada uno de estos nutrientes según la clasificación Nova, se encontró que el 65.8% de los PUP presentaron exceso de azúcar libre, 53.4% de grasa total, 59.0% de grasa saturada, 5.1% de grasa trans, 43.7% de sodio y 18.6% contenían edulcorantes (datos no mostrados).

La mayoría de la población consumió diariamente al menos un AP y/o PUP identificado como excesivo en algún nutriente crítico relacionado con las EC (según el MPN de la OPS), de la siguiente manera: 73.3% con contenido excesivo en azúcar libre, 75.2% en grasa total, 77.0% en grasa saturada, 8.4% en grasa trans, 83.9% en sodio y 36.8% contenía edulcorantes ( Tabla 3 ).

**Tabla 3:** Prevalencia de consumo de productos definidos como excesivos en nutrientes priorizados por OPS según MPNa

Tipo de productos y alimentos	Azúcar Libre		Grasa Total		Grasas Saturadas		Grasas Trans		Sodio		Edulcorantes	
					
	n	% (IC 95%)	n	% (IC 95%)	n	% (IC 95%)	n	% (IC 95%)	n	% (IC 95%)	n	% (IC 95%)
Solo alimentos que no se aplican al MPN de la OPS^b^	330	7.5 (6.8–8.4)	330	7.5 (6.8–8.4)	330	7.5 (6.8–8.4)	330	7.5 (6.8–8.4)	330	7.5 (6.8–8.4)	330	7.5 (6.8–8.4)
Productos definidos como no excesivos en nutrientes críticos relacionados con las ENT según el MPN de la OPS^c^	839	19.2 (18.0–20.3)	759	17.3 (16.2–18.5)	679	15.5 (14.5–16.6)	3,68	84.0 (82.9–85.1)	376	8.6 (7.8–9.5)	2,442	55.7 (54.3–57.2)
Productos definidos como excesivos en nutrientes críticos relacionados con las ENT según el MPN de la OPS^d^												
1 producto	1,020	23.3 (22.1–24.6)	1,102	25.2 (23.9–26.5)	1,008	23.0 (21.8–24.3)	311	7.1 (6.4–7.9)	1,113	25.4 (24.1–26.7)	461	10.5 (9.7–11.5)
2 productos	796	18.2 (17.1–19.3)	841	19.2 (18.1–20.4)	864	19.7 (18.6–20.9)	51	1.2 (0.1–1.5)	965	22.0 (20.8–23.3)	467	10.7 (9.8–11.6)
3 productos	559	12.8 (11.8–13.8)	606	13.8 (12.8–14.9)	621	14.2 (13.2–15.2)	7	0.2 (0.1–0.3)	661	15.1 (14.1–16.2)	328	7.5 (6.7–8.3)
≥ 4 productos	838	19.1 (18.0–20.3)	744	17.0 (15.9–18.1)	880	20.1 (18.9–21.3)	1	0.0 (0.0–0.2)	937	21.4 (20.2–22.6)	354	8.1 (7.3–8.9)

IC 95%: intervalo de confianza del 95%; EC: enfermedades crónicas asociadas a la alimentación; MPN-OPS: Modelo de Perfil de Nutrientes de la Organización Panamericana de la Salud.

^a^Consumo de alimentos. Perfil Alimentario y Nutricional de Antioquia 2019. n = 4,382 personas

^b^Alimentos que no aplican al MPN de la OPS: Alimentos naturales o mínimamente procesados e ingredientes culinarios procesados.

^c^Alimentos que aplican al MPN de la OPS, pero sin alto contenido de nutrientes relacionados con las EC según el MPN de la OPS (para azúcares libres < 10% de la energía total, para grasas totales < 30 % de la energía total, para grasas saturadas < 10% de energía total, para grasas trans < 1% de la energía total, para sodio < 1 mg por kcal).

^d^Productos con contenido excesivo de nutrientes relacionados con las ENT según MPN de la OPS (para azúcares libres ≥ 10 % de la energía total, para grasas totales ≥ 30% de la energía total, para grasas saturadas ≥ 10% de la energía total, para grasas trans ≥ 1% de la energía total, para sodio ≥ 1 mg por kcal).

En cuanto al consumo de AP y/o PUP sin un contenido excesivo de nutrientes críticos, se encontró que el 19.2%, 17.3% y 15.5% de las personas consumieron productos sin contenido excesivo de azúcares libres, grasa total y grasas saturadas, respectivamente. En el caso de las grasas trans y el sodio, la proporción de personas que consumió productos sin exceso fue de 84.0% y 8.6%, respectivamente. De otro lado, una proporción aún menor de antioqueños (7.5%) consumió únicamente alimentos e ingredientes culinarios sin procesar y mínimamente procesados ( Tabla 3 ).

Al analizar el aporte calórico proveniente de los nutrientes críticos, se encontró que en la dieta del total de la población antioqueña el 16.7% de las calorías totales provienen de los azúcares libres, 27.1% de las grasas totales, 11.5% de las grasas saturadas, 0.21% de las grasas trans. El contenido promedio de sodio en la dieta total (sin tomar en cuenta el consumo de sal de mesa) fue de 897.6 mg en adultos y adolescentes, 983.2 mg en niños 5–10 años, y de 840.5 mg en los niños menores de 5 años. En cuanto a la cantidad de edulcorantes, el consumo promedio al día fue de 0.98, siendo en todos los casos, mayor en el grupo que consume productos con contenido excesivo en nutrientes críticos, comparado con aquellos que consumen productos sin contenido excesivo ( Tabla 4 ).

**Tabla 4:** Contenido promedio de nutrientes priorizados por OPS según el MPN en la dieta diaria total de la población y en dos fracciones de la poblacióna

Contenido de nutrientes en la dieta	Toda la población	Fracciones de la población con dieta compuesta por productos		Coef. (95%CI) sin ajustar^d^	Coef. (95%CI) ajustado^e^

		Con un contenido excesivo de nutrientes priorizados por OPS^b^	Sin contenido excesivo de nutrientes priorizados por OPS^c^		
			
Contenido dietético de nutrientes	Media (IC 95%)	Media (IC 95%)	Media (IC 95%)		
Azúcar libre (% de la ingesta total de energía)	16.7 (16.3–17.0)	16.8 (16.4–17.1)	16.4 (15.6–17.1)	0.4 (-0.3–1.3)	0.8 (0.1–1.5)
Grasa total (% de la ingesta total de energía)	27.1 (26.9–27.4)	28.7 (28.4–29.0)	22.45 (21.9–23.0)	6.2 (5.6–6.8)	5.8 (5.3–6.4)
Grasa saturada (% de la ingesta total de energía)	11.5 (11.3–11.6)	12.4 (12.2–12.5)	8.42 (8.2–8.7)	3.9 (3.65–4.21)	3.8 (3.5–4.1)
Grasa trans (% de la ingesta total de energía)	0.2 (0.2–0.2)	0.4 (0.3–0.4)	0.2 (0.2–0.2)	0.2 (0.2–0.2)	0.2 (0.2–0.2)
Sodio mg) Adultos y adolescentes	897.6 (866.3–928.9)	1,029.7 (996.7–1,062.6)	290.2 (219.4–360.9)	739.5 (661.5–817.5)	698.7 (620.8–776.6)
Sodio (mg) Niños entre 5 y 10 años	983.2 (922.1–1,044.3)	1,044.2 (983.2–1,105.3)	361.1 (166.3–555.9)	683.1 (479.0–887.3)	659.2 (452.4–865.9)
Sodio (mg) Niños < 5 años	840.5 (788.5–892.6)	872.2 (820.2–924.1)	353.6 (149.7–557.4)	518.6 (308.3–728.9)	498.0 (289.0–707.0)
Edulcorantes (cantidad/día)	0.98 (0.93–1.03)	2.68 (2.59–2.77)	0	2.68 (2.61–2.75)	2.67 (2.60–2.74)

IC 95%: Intervalo de Confianza del 95%; EC: Enfermedades crónicas asociadas a la alimentación; MPN-OPS: Modelo de Perfil de Nutrientes de la Organización Panamericana de la Salud.

^a^Consumo de alimentos. Perfil Alimentario y Nutricional de Antioquia 2019. n = 4,382 personas

^b^Productos con contenido excesivo de nutrientes relacionados con las EC según MPN de la OPS (para azúcares libres ≥ 10% de la energía total, para grasas totales ≥ 30% de la energía total, para grasas saturadas ≥ 10% de la energía total, para grasas trans grasas ≥ 1% de la energía total, para sodio ≥ 1 mg por kcal).

^c^Alimentos que no aplican al MPN de la OPS (alimentos naturales o mínimamente procesados e ingredientes culinarios procesados) y alimentos a los que aplica el MPN de la OPS, pero que no tienen un contenido excesivo de nutrientes relacionados con las EC (para azúcares libres < 10% de la energía total, para grasas totales < 30% de la energía total, para grasas saturadas < 10% de energía total, para grasas trans < 1% de energía total, para sodio < 1 mg por kcal).

^d^Modelos de regresión lineal sin ajustar.

^e^Modelos de regresión lineal ajustados por grupos de edad, sexo, nivel educativo y zona de residencia.

Al analizar los modelos crudos y ajustados (por variables sociodemográficas) para cada nutriente crítico, se encontró que en todos hay diferencias significativas, dado que el coeficiente del porcentaje promedio (azúcar libre, grasa total, grasa saturada y grasa trans), cantidad consumida de mg (sodio) y la cantidad/día (edulcorantes) fue mayor en el grupo que consumió productos con exceso en nutrientes críticos ( Tabla 4 ).

La Tabla 5 expone las fracciones de la población según la composición de la dieta, así: población con una dieta con productos con contenido excesivo en nutrientes críticos, frente a aquellos que no tenían consumo de estos productos. Para el caso de la población que consumía productos con contenido excesivo en azúcar libre, grasa saturada, grasa total, sodio y grasas trans, se encontró que el 75.5%, 71.3%, 42.6%, 10.9% y 3.5%, respectivamente, superó el límite superior recomendado para estos nutrientes. Mientras que, en la población con una dieta sin productos con contenido excesivo en nutrientes críticos, el porcentaje de la población que no cumplió con la recomendación de ingesta fue menor: 63.7% para el azúcar libre, 29.8% para las grasas saturadas y 19.4% para la grasa total.

**Tabla 5:** Prevalencia de niveles de ingesta no recomendados de nutrientes priorizados por OPSa en la población total y por fracciones de la población con una dieta con y sin productos con un contenido excesivo de estos nutrientes críticos según modelo de perfil de nutrientes de la OPSb

Nutriente crítico relacionado con las EC	Toda la población	Fracciones de la población con dieta compuesta por productos		RP (IC 95%)^e^	
		Con un contenido excesivo de nutrientes priorizados por OPS^c^	Sin contenido excesivo de nutrientes priorizados por OPS^d^		
			
	Porcentaje de individuos que superaron las metas de ingesta recomendadas por la OMS (%)			Cruda	Ajustada^f^

	Media (IC 95%)	Media (IC 95%)	Media (IC 95%)		
Azúcar libre	72.3 (71.0–73.7)	75.5 (74.0–77.0)	63.7 (60.8–66.4)	1.2 (1.1–1.2)	1.15 (1.1–1.2)
Grasa total	36.9 (35.4–38.3)	42.6 (41.0–44.3)	19.4 (17.1–21.8)	2.2 (1.9–2.5)	2.0 (1.7–2.2)
Grasas saturadas	61.7 (60.3–63.7)	71.3 (69.7–72.8)	29.8 (27.1–32.7)	2.4 (2.2–2.6)	2.2 (2.0–2.5)
Grasas trans	0.7 (0.5–1.0)	3.6 (2.1–6.0)	0.4 (0.2–0.7)	8.8 (4.3–18.2)	8.2 (3.9–17.2)
Sodio	9.2 (8.4–10.1)	10.9 (9.9–11.9)	0.6 (0.2–1.5)	19.2 (7.2–51.3)	15.3 (5.7–40.9)
Edulcorantes	36.7 (35.3–38.2)	.	.	1.6 (1.6–1.7)	1.6 (1.6–1.7)

IC 95%: Intervalo de Confianza del 95%; EC: Enfermedades crónicas asociadas a la alimentación; MPN-OPS: Modelo de Perfil de Nutrientes de la Organización Panamericana de la Salud.

^a^Contenido de nutrientes críticos relacionados con las ENT: azúcares libres, grasas totales, grasas saturadas y grasas trans (% de la ingesta total de energía). Contenido de sodio: sodio total (mg) menos el valor recomendado por grupo de edad [2000 mg (para adultos y adolescentes); 1640mg (para niños de 5-10 años); y 1122 mg (para niños menores de 5 años)]. Pauta: ingesta de sodio para adultos y niños. Organización Mundial de la Salud; 2012; Requerimientos energéticos humanos. FAO/OMS/UNU; 2004

^b^Consumo de alimentos. Perfil Alimentario y Nutricional de Antioquia 2019. n = 4.382 personas

^c^Productos con contenido excesivo de nutrientes relacionados con las EC según MPN de la OPS (para azúcares libres ≥ 10% de la energía total, para grasas totales ≥ 30% de la energía total, para grasas saturadas ≥ 10% de la energía total, para grasas trans grasas ≥ 1% de la energía total, para sodio ≥ 1 mg por kcal).

^d^Alimentos que no aplican al MPN de la OPS (alimentos naturales o mínimamente procesados e ingredientes culinarios procesados) y alimentos a los que aplica el MPN de la OPS, pero que no tienen un contenido excesivo de nutrientes relacionados con las EC (para azúcares libres < 10% de la energía total, para grasas totales < 30% de la energía total, para grasas saturadas < 10% de energía total, para grasas trans < 1% de energía total, para sodio < 1 mg por kcal).

^e^Coeficientes de los modelos de regresión logística.

^f^Ajustado grupos de edad, sexo, nivel educativo y zona de residencia.

Asimismo, en la Tabla 5 se observan las razones de prevalencia ajustadas, indicando que la fracción de la población que consumió productos con un contenido excesivo en nutrientes críticos tiene un incremento significativo en la posibilidad de inadecuación en el consumo de azúcares libres, grasa total, grasas saturadas, grasas trans, sodio y edulcorantes, en comparación con la fracción de la población que no consumió productos con un contenido excesivo de nutrientes críticos (la razón de prevalencia varía entre 1.15 (IC 95% 1.1–1.2) para el azúcar libre y 15.3 (IC 95% 5.7–40.9) para el sodio.

## DISCUSIÓN

Esta investigación evidencia que cerca de la mitad de los AP y PUP consumidos por la población antioqueña tenían exceso en al menos un nutriente crítico. Los resultados son similares a estudios realizados en Colombia, México y Honduras^20^. La mayoría de los sujetos consumieron diariamente uno o más de estos productos. Además, se destaca que quienes consumieron AP y/o PUP con cantidades excesivas en algún nutriente crítico tuvieron una dieta con peor calidad nutricional y con la mayor posibilidad de inadecuación en el consumo de los nutrientes críticos y edulcorantes relacionados con las EC (incumplimiento recomendaciones ingesta OMS).

De otro lado, una proporción menor de individuos consumió productos con exceso de grasa trans, situación que puede explicarse en parte por las regulaciones colombianas para este componente, como son: el artículo 8 de la ley 1355 de 2009^23^, la resolución 2508 de 2012^24^y la resolución 2154 de 2012, expedidas por el Ministerio de Salud y Protección Social^25^.

En correspondencia con las evidencias, al implementarse el etiquetado frontal de advertencia (basado en el perfil de nutrientes de la OPS), la mayoría de los AP y PUP serán candidatos a llevar por lo menos una etiqueta y/o ser gravados con un impuesto saludable, esto, con el propósito de reducir su consumo y consecuencias en salud^26^.

Las dietas de personas que consumían productos con un contenido excesivo de nutrientes críticos contenían significativamente más grasas totales (> 5.8%), más grasas saturadas (> 3.8%), y una cantidad mayor de sodio (> 498 mg), en comparación con las dietas de personas que no consumían estos productos. Los resultados concuerdan con la investigación de Machado y colaboradores^26^. En el caso del azúcar libre, se presentaron diferencias menores, posiblemente porque los antioqueños tienen un alto consumo de alimentos naturales y/o mínimamente procesados con alto contenido de azúcar libre como panela, miel y jugos naturales con adición de azúcar, lo que podría indicar que el aporte del azúcar libre en la dieta proviene de diferentes fuentes.

Por otra parte, es importante mencionar que el MPN de la OPS también recomienda advertir cuando los AP y PUP contienen cualquier cantidad de edulcorantes (artificiales, naturales, calóricos y no calóricos). Aunque se identificó la mayor proporción de edulcorantes que consumían los individuos con ingesta de AP y PUP (2.7 edulcorantes más), se desconoce la cantidad exacta de edulcorantes que consume la población antioqueña en su dieta (gramos o miligramos), debido a que la industria no está obligada a declarar la cantidad (sólo informan su presencia en la lista de ingredientes); asimismo, las evidencias disponibles no son concluyentes sobre sus efectos benéficos o perjudiciales en la salud^29^.

Este estudio evidenció que la ingesta de productos con contenido excesivo de nutrientes críticos explica la inadecuación en la dieta de la población, de acuerdo a las recomendaciones de la OMS. Este resultado es similar a los encontrados en estudios realizados en Australia^26^y Uruguay^27 , 28^.

La presente investigación soporta las medidas de políticas públicas establecidas en el departamento de Antioquia, a través del Plan Docenal de Seguridad Alimentaria y Nutricional, en términos de reducir el consumo de PUP a menos del 10.0% de la energía total consumida para el 2031^30^, así como las políticas nacionales y propuestas para implementar un etiquetado frontal de advertencia octagonal negro (con la palabra exceso), según las recomendaciones del perfil de nutrientes de la OPS^18^; además, da bases sólidas a la implementación de impuestos saludables a los PUP.

Como fortalezas de este estudio se destacan: la calidad de la base de datos y el importante número de registros recolectados a través del recordatorio de 24 horas, el cual fue efectuado por nutricionistas dietistas estandarizados en la técnica; la representatividad de la muestra (nivel departamental), por grupos de edad, sexo, nivel educativo y zona de residencia; y la modelación de las posibilidades diferenciales de inadecuación en la dieta. Es el primer estudio en Antioquia que evalúa el consumo de nutrientes priorizados por OPS, así como el contenido de edulcorantes en la dieta, siendo estos resultados la línea de base para las medidas de políticas públicas locales y nacionales (regulación de los AP y PUP).

En relación a las limitaciones, nuestro estudio se basó en solo un recordatorio de 24 horas, por lo que es posible que en algunos casos no represente el consumo habitual^17^. En cuanto a la ingesta de sodio, es probable que esté subestimada, debido a que en el PANA no se incluyó la sal discrecional, añadida por las personas en los alimentos preparados en sus casas o en restaurantes. De otro lado, el instrumento de recordatorio de 24 horas empleado en el PANA no indaga por la marca de los AP y PUP consumidos por cada individuo, lo que dificulta conocer con plena certeza el contenido de algunos de los nutrientes críticos (principalmente azúcares libres, grasas trans y edulcorantes, estos últimos, en lista de ingredientes) que no están dispuestos en las tablas de composición de alimentos; aun así, esta limitante se trató de subsanar, en el caso de los azúcares libres aplicando el panel D del modelo de perfil de nutrientes de la OPS, y con las grasas trans y edulcorantes, rastreando la información directamente en las etiquetas de productos similares a los reportados en la base de datos y/o consultando la base de datos de la USDA.

## CONCLUSIÓN

La población antioqueña que consume AP y PUP con cantidades excesivas de azúcares libres, grasas totales, grasas saturadas, sodio y/o edulcorantes, presenta una dieta desbalanceada. Reducir el consumo de estos productos y volver a una dieta natural y/o mínimamente procesada puede ser una estrategia efectiva para alcanzar las recomendaciones de ingesta de nutrientes priorizados por OPS en la población antioqueña.
